# volBrain: An Online MRI Brain Volumetry System

**DOI:** 10.3389/fninf.2016.00030

**Published:** 2016-07-27

**Authors:** José V. Manjón, Pierrick Coupé

**Affiliations:** ^1^Instituto de Aplicaciones de las Tecnologías de la Información y de las Comunicaciones Avanzadas (ITACA), Universitat Politècnica de ValènciaValencia, Spain; ^2^Pictura Research Group, Unité Mixte de Recherche Centre National de la Recherche Scientifique (UMR 5800), Laboratoire Bordelais de Recherche en Informatique, Centre National de la Recherche ScientifiqueTalence, France; ^3^Pictura Research Group, Unité Mixte de Recherche Centre National de la Recherche Scientifique (UMR 5800), Laboratoire Bordelais de Recherche en Informatique, University BordeauxTalence, France

**Keywords:** MRI, brain, segmentation, multi-atlas label fusion, cloud computing

## Abstract

The amount of medical image data produced in clinical and research settings is rapidly growing resulting in vast amount of data to analyze. Automatic and reliable quantitative analysis tools, including segmentation, allow to analyze brain development and to understand specific patterns of many neurological diseases. This field has recently experienced many advances with successful techniques based on non-linear warping and label fusion. In this work we present a novel and fully automatic pipeline for volumetric brain analysis based on multi-atlas label fusion technology that is able to provide accurate volumetric information at different levels of detail in a short time. This method is available through the volBrain online web interface (http://volbrain.upv.es), which is publically and freely accessible to the scientific community. Our new framework has been compared with current state-of-the-art methods showing very competitive results.

## Introduction

Automated and reliable quantitative MRI-based brain image analysis has a huge potential to objectively help in the diagnosis and follow-up of many neurological diseases. Specifically, MRI brain structure volumetry is being increasingly used to understand the nature and evolution of those diseases.

For many years manual segmentation has been the method of choice to accurately analyze specific brain structures. However, this task is tedious and time consuming, limiting its use in clinical practice. To help in the quantification process, tools have been proposed making the brain segmentation problem one of the most intensively studied topics during the last years. The increased amount of neuroimaging data to process and the increasing complexity of the methods to analyze those challenges image processing methods. This motivates the development of innovative approaches able to address challenges related to this new “Big Data” paradigm (Van Horn and Toga, 2014). Efficient, automatic, robust and reliable methods for automatic brain analysis will play a major role in near future, most of them powered by cost-effective cloud-based solutions.

The brain segmentation problem has been studied at different scales (from macroscopic tissues to local structures). One of the first neuroimaging analysis tasks has been the segmentation of the brain parenchyma in order to separate it from non-brain tissues and compute brain volume. This brain extraction operation, also called skull stripping or intracranial cavity (ICC) extraction depending on the definition of the volume segmented [typically depending on the inclusion or not of external CerebroSpinal Fluid (CSF)]. The BET (Brain Extraction Tool) software from the FSL image processing library (Smith, 2002) is one a well-known and widely used brain extraction techniques. Other techniques such as Brain Surface Extractor (BSE) have been also used successfully (Sandor and Leahy, 1997). More recently, multi-atlas label fusion methods have been shown to be competitive (Leung et al., 2011; Eskildsen et al., 2012). Intracranial cavity extraction can also be obtained indirectly as part of the full modeling of brain intensities using a parametric models as done in Statistical Parametric Mapping (SPM) (Ashburner and Friston, 2005) or VBM8 (Nenadic et al., 2010) software packages. Recently, we presented a novel approach for intracranial cavity extraction called NICE (Manjón et al., 2014) which is an evolution of the BEaST technique enabling faster and more accurate results.

Another set of methods aim at classifying the main intracranial tissues such as white matter (WM), gray matter (GM), and CSF. A usual approach is to model the histogram of the ICC area using a mixture of Gaussians estimated with the EM algorithm (Wells et al., 1996) or with fuzzy C-means clustering (Ahmed et al., 2002). A common feature of those methods is the use of *a priori* information in the form of spatial probability maps (e.g., SPM software Ashburner and Friston, 2005). All these methods assign a membership degree or probability to belong to specific tissue to every voxel rather than calculate the actual amount of each tissue within each voxel. For this reason some authors used the concept of partial volume coefficients (PVC) to represent the actual amount of every tissue within each voxel (Tohka et al., 2004; Manjón et al., 2010a).

Although, the global amount of WM, GM, and CSF within the ICC may be an interesting biomarker for quantitative brain analysis, some diseases present early local alterations instead of global ones. Therefore, the analysis of different brain structures separately can be very useful. In addition, the assessment of brain structure asymmetries may be also interesting to study normal/abnormal brain development and to detect alterations due to some neurological diseases. Segmentation of structures such us cerebrum, cerebellum, brainstem, and brain hemispheres is thus of interest to assess brain asymmetry. Several automatic strategies have been developed for hemisphere and compartmental segmentation. First attempts were based on mid-sagittal plane extraction or linear registration (Brummer, 1991; Sun and Sherrah, 1997; Prima et al., 2002) but it was shown that these approaches may produce inaccurate segmentation results because the brain could be asymmetric (Zhao et al., 2010). Current state of the art hemisphere/compartmental segmentation methods are based on nonlinear registration (Maes et al., 1999; Larsson, 2001) or on structure-reconstruction. In the latter, seed voxels representing the hemispheres (and cerebellum) are identified before hemispheres can be reconstructed (Hata et al., 2000; Mangin et al., 2004; Zhao et al., 2010). Recently, we presented a novel and competitive approach for compartmental segmentation called NABS (Romero et al., 2015) that is based on multi-atlas technology using non-local label fusion (Coupé et al., 2011).

Finally, it may be also interesting to measure local volumes at a finer scale since many pathologies affect specific areas of the brain. For instance, the volumes of the hippocampi and the lateral ventricles have been shown to be early biomarkers of Alzheimer disease (Coupé et al., 2012). To segment the subcortical nuclei, several automatic methods have been proposed using deformable models (Shen et al., 2002; Chupin et al., 2007) or atlas/template-warping techniques (Collins et al., 1995; Barnes et al., 2008). More recently, multi-atlas label fusion segmentation techniques has gained in popularity because they can combine multiple atlas information, thereby minimizing mislabeling from inaccurate affine or non-linear registration (Rohlfing et al., 2004; Heckemann et al., 2006; Collins and Pruessner, 2010; Lötjönen et al., 2010). The non-local label fusion method proposed by Coupé et al. (2011) addresses this problem in an accurate and efficient implementation only requiring a fast linear registration.

Several recent software tools have been developed to automatically obtain some or all of these volumetric measures using different strategies. For example, the SPM software is a widely used tool to analyze global GM or WM alterations. Voxel-Based Morphometry (VBM) toolbox (an extension of SPM) has also been used to measure local GM atrophy. To perform more specific volume measurements tools like the FSL package (Jenkinson et al., 2012) or Freesurfer (Fischl et al., 2002) are freely available. FSL is a comprehensive library of analysis tools for functional MRI, anatomical MRI and DTI brain imaging data. One of these tools, called FIRST (Patenaude et al., 2011), is able to automatically segment subcortical brain structures. Similarly, the Freesurfer pipeline can be used for volumetric segmentation, cortical surface reconstruction and cortical parcellation; it has been used in numerous studies despite its high computational burden due to its ease of use. The great success of these tools is due to their success in obtaining volumetric information from MRI data, but also because of their free public availability.

The aim of this paper is to present volBrain, a new software pipeline for volumetric brain analysis. This pipeline provides automatically volumetric brain information at different scales in a very simple web-based interface not requiring any installation or advanced computational requirements. In the following sections, the different parts of the volBrain platform are described and some performance evaluation presented by comparing results to existing methods.

## Materials and methods

The volBrain system provides volumes/segmentations and structure asymmetry ratios at different scales:

- Intracranial cavity (ICC was defined as the sum of all WM, GM, and cerebrum-spinal fluid (CSF)).
- Tissue Volumes: WM, GM, and CSF volumes.
- Cerebrum, cerebellum, and brainstem volumes (separating left from right cerebrum and cerebellum).
- Lateral ventricles and subcortical GM structures (putamen, caudate, pallidum, thalamus, hippocampus, amygdala, and accumbens).

All these segmentations with the exception of tissue volumes are based on different adaptations of multi-atlas patch-based label fusion segmentation (Coupé et al., 2011). The proposed pipeline is based on a library of manually labeled cases to perform the segmentation process. We will first describe the template library construction and then the full segmentation pipeline. Finally, the volBrain web interface will be presented.

### Template library construction

#### Library dataset description

The library of manually labeled templates was constructed using subjects from different public available datasets. To include a wide range of age, different datasets covering nearly the entire human life-span were used. Images were downloaded from the different websites in raw format without any preprocessing. MRI data from the following databases were used:

- **Normal adults dataset**: Thirty normal subjects (age range: 24–75 years) were randomly selected from the open access IXI dataset (http://www.brain-development.org). This dataset contains images of nearly 600 healthy subjects from several hospitals in London (UK). Both 1.5 T (7 cases) and 3 T (23 cases) images were included in our training dataset. 3T images were acquired on a Philips Intera 3T scanner (*TR* = 9.6 ms, *TE* = 4.6 ms, flip angle = 8°, slice thickness = 1.2 mm, volume size = 256 × 256 × 150, voxel dimensions = 0.94 × 0.94 × 1.2 mm^3^). 1.5 T images were acquired on a Philips Gyroscan 1.5T scanner (*TR* = 9.8 ms, *TE* = 4.6 ms, flip angle = 8°, slice thickness = 1.2 mm, volume size = 256 × 256 × 150, voxel dimensions = 0.94 × 0.94 × 1.2 mm^3^).
- **Alzheimer Disease (AD) dataset**: Ten patients with Alzheimer's disease (age range = 75–80 years, MMSE = 23.7 ± 3.5, CDR = 1.1 ± 0.4) scanned using a 1.5 T General Electric Signa HDx MRI scanner (General Electric, Milwaukee, WI) were selected from OASIS dataset. This dataset consisted of high resolution T1-weighted sagittal 3D MP-RAGE images (*TR* = 8.6 ms, *TE* = 3.8 ms, *TI* = 1000 ms, flip angle = 8°, matrix size = 256 × 256, voxel dimensions = 0.938 × 0.938 × 1.2 mm^3^). These images were downloaded from the brain segmentation testing protocol website (https://sites.google.com/site/brainseg/) although they belong originally to the open access OASIS dataset (http://www.oasis-brains.org).
- **Pediatric dataset**: Ten infant cases were also downloaded from the brain segmentation testing protocol (Kempton et al., 2011) website (https://sites.google.com/site/brainseg). These data were originally collected by Gousias et al. (2008) and are also available at http://www.brain-development.org. The selected 10 cases are from the full sample of Thirty-two 2-year old infants born prematurely (age = 24.8 ± 2.4 months). Sagittal T1 weighted volumes were acquired from each subject (1.0 T Phillips HPQ scanner, *TR* = 23 ms, *TE* = 6 ms, slice thickness = 1.6 mm, matrix size = 256 × 256, voxel dimensions = 1.04 × 1.04 × 1.6 mm^3^ resliced to isotropic 1.04 mm^3^).

#### Preprocessing

To generate the templates library, all 50 selected T1-weighted images were preprocessed using the following steps:

1. **Denoising:** All images were denoised using the Spatially Adaptive Non-Local Means (SANLM) Filter (Manjón et al., 2010b) to enhance the image quality. The SANLM filter can deal with spatially varying noise levels across the image without explicitly estimating the local noise level which makes it ideal to process data with either stationary or spatially varying noise in a fully automatic manner. This method has been included in several software packages already such as VBM8, CAT12 (http://www.neuro.uni-jena.de/vbm) or the Connectome Computation System (Xu et al., 2015).
2. **Coarse Inhomogeneity correction:** To further improve the image quality, an inhomogeneity correction step was applied using the N4 method (Tustison et al., 2010). The N4 method is recent and more efficient and robust improvement of the N3 method (Sled et al., 1998), implemented as part of the ITK toolbox (Ibáñez et al., 2003).
3. **MNI space registration:** The template library and the subject to be segmented have to be located in the same stereotactic space. A spatial normalization based on a linear affine registration to the Montreal Neurological Institute (MNI152) space was performed using ANTS software (Avants et al., 2009). The resulting images in the MNI space have a size of 181 × 217 × 181 voxels with 1 mm^3^ voxel resolution. The transformation matrix was estimated using the inhomogeneity corrected image in previous step but applied the denoised image without IH correction. Although, N4 removes most of the inhomogeneity it sometimes does not remove it completely (especially on 3T cases). Therefore, N4 was used only to improve the linear registration parameter estimation.
4. **Fine Inhomogeneity correction:** Once the data is in MNI space we used the inhomogeneity correction capabilities of SPM8 (Ashburner and Friston, 2005) toolbox. We found this model-based method to be quite robust once the data were in the MNI space (especially for 3T images).
5. **Intensity normalization:** As the proposed method is based on the estimation of image similarities using intensity-derived measures, every image in the library was normalized. We used a tissue-derived approach to force mean intensities of WM, GM, and CSF to be as similar as possible across subjects of the library in a similar manner than Lötjönen et al. (2010). Mean values of CSF, GM, and WM tissues were estimated using the Trimmed Mean Segmentation (TMS) method (Manjón et al., 2008) that robustly estimates the mean values of the different tissues by excluding partial volume voxels from the estimation process and by using an unbiased robust mean estimator. Finally, a piecewise linear intensity mapping (Lötjönen et al., 2010) was applied ensuring that WM had an average intensity of 250, GM of 150 and CSF of 50.

#### Manual labeling

Manual labeling at different scales was performed by a trained expert using ITK-SNAP software. Details of intracranial cavity mask and macrostructures (cerebrum, cerebellum, and brainstem) can be found in the corresponding original papers (Manjón et al., 2014; Romero et al., 2015). Lateral ventricles and subcortical structures were manually segmented from scratch using ITK-SNAP software using the 3 orthogonal views to avoid any inconsistency in 3D. Lateral ventricles label were thresholded using a threshold of 100 over the intensity-normalized images to get a consistent label definition (note that choroid plexus was not included in our lateral ventricles definition). All subcortical structures were segmented according to the current common definition criteria with the exception of hippocampus that was segmented using EADC protocol (Frisoni and Jack, 2011).

We further increased the number of available priors in the library by flipping them along the mid-sagittal plane using the symmetric properties of the human brain. Therefore, a total number of 100 labeled training templates (original and flipped) have been created as done in BEaST paper (Eskildsen et al., 2012).

### volBrain pipeline description

The volBrain pipeline is a set of image processing tasks that aims at improving the quality of the input images and to set them into a specific geometric and intensity space (the same than the used one for the manually labeled training templates) to later segment the different structures/tissues of interest. The volBrain pipeline is based on the following steps:

1. Spatially adaptive Non-local means denoising
2. Rough inhomogeneity correction
3. Affine registration to MNI space
4. Fine SPM based inhomogeneity correction
5. Intensity normalization
6. Non-local Intracranial Cavity Extraction (NICE)
7. Tissue classification
8. Non-local hemisphere segmentation (NABS)
9. Non-local subcortical structure segmentation

**Figure 1:**
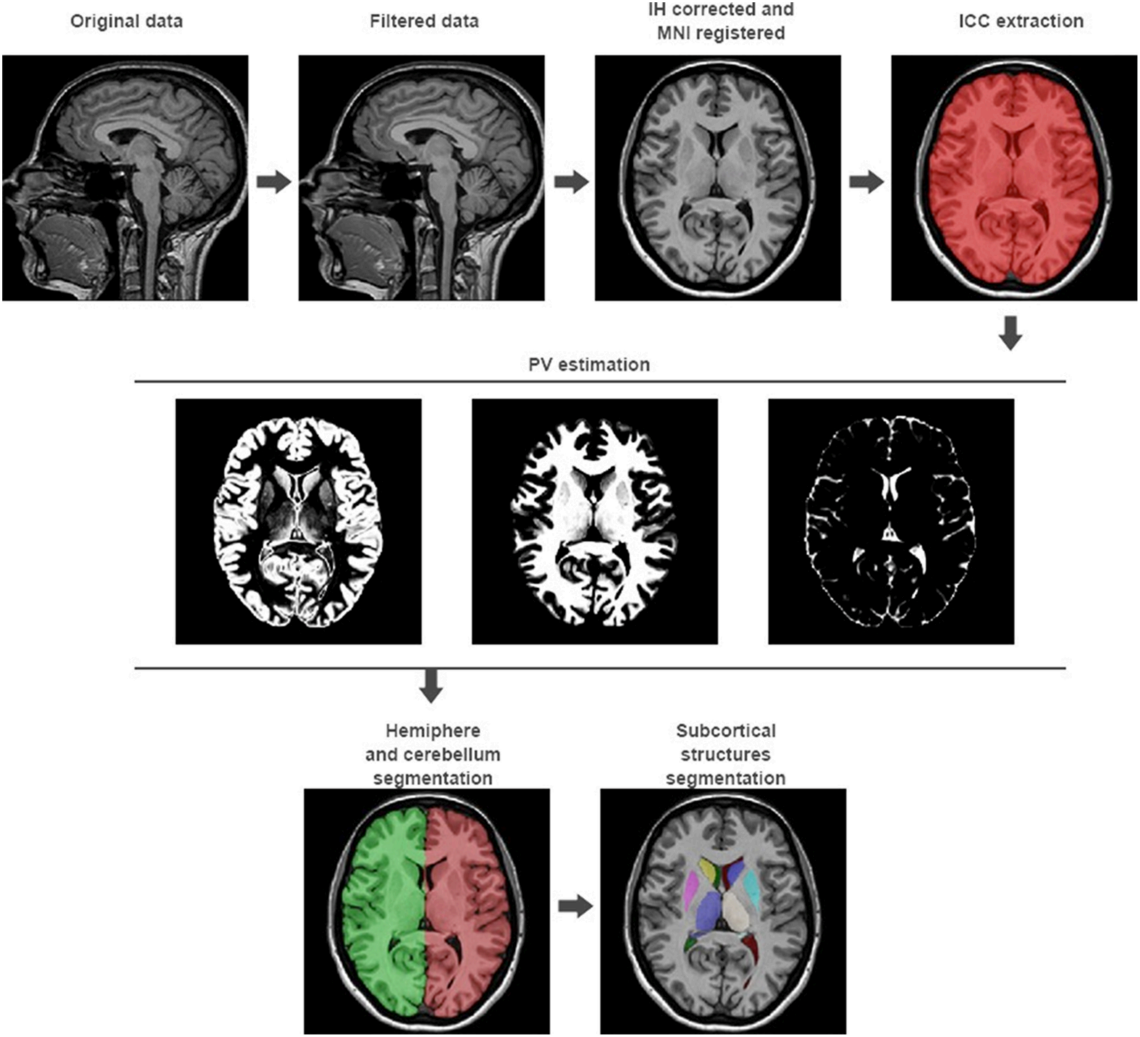
**volBrain processing pipeline**. In the first row, the preprocessing of any new subject is presented. It consists in a non-local noise reduction filter, inhomogeneity correction, MNI space registration, intensity normalization, and ICC extraction. In the second row, the result of the global tissue estimation (GM, WM, and CSF) is shown. In the third row, the result of the macrostructures and subcortical structures segmentation is presented.

Steps from 1 to 5 represent the preprocessing to be applied to the input images to set them in the same geometric and intensity space than the template library. Steps from 6 to 9 are focused in the estimation of different brain volumes at different scales (see Figure 1). We will now describe them in detail.

#### Non-local intracranial cavity extraction (NICE)

NICE method is based on a multi-scale non-local label fusion scheme and it represents an evolution of BEAST method (Eskildsen et al., 2012) to improve both accuracy and reproducibility, and to significantly reduce the computational burden of the method. Furthermore, NICE method intracranial cavity (ICC) mask definition includes WM, GM, and all CSF (both internal and external) that is a very important confound factor in brain analysis. Details of NICE method can be found in Manjón et al. (2014).

#### Tissue classification

Once the ICC is segmented, only WM, GM, and CSF voxels are included within the ICC mask. To obtain the tissue proportions we used an intensity driven approach. As done for intensity normalization, mean values of CSF, GM, and WM tissues were estimated using the TMS method (Manjón et al., 2008). TMS robustly estimates the mean values of the different tissues by excluding partial volume voxels from the estimation jointly with the use of an unbiased robust mean estimator. Finally, the PVC and the crisp segmentation were computed using the estimated mean values. Details of this method can be found in Manjón et al. (2008).

#### Non-local automatic brain hemisphere segmentation (NABS)

NABS method is also based on a multi-scale non-local label fusion scheme. This method splits the GM and WM from ICC mask into five regions: left-cerebrum, right-cerebrum, left-cerebellum, right-cerebellum, and brainstem. NABS is able to rapidly separate all this regions by only processing the so-called “uncertain” areas. Details of this method can be found in Romero et al. (2015).

#### Non-local subcortical structure segmentation

Subcortical structure segmentation was performed using an updated version of the algorithm described in Coupé et al. (2011). As proposed by Coupé et al., voxel labeling is performed using a weighted label vote scheme based on the non-local means estimator (Buades et al., 2005). This technique is generally called non-local label fusion in the literature.

In brief, for all voxels *x*_*i*_ of the image to be segmented, the estimation of the final label is based on a weighted label fusion *v(x*_*i*_*)* of all labeled samples in the selected library (i.e., inside the search area *V*_*i*_ for the N considered subjects):

(1) $$v(x_{i})=\frac{\sum_{s=1}^{N}\sum_{j\in V_{i}}w(x_{i},x_{s,j}).y_{s,j}}{\sum_{s=1}^{N}\sum_{j\in V_{i}}w(x_{i},x_{s,j})},$$

where *y*_*s, j*_ is the label given by the expert to voxel *x*_*s, j*_ at location *j* in subject *s*. The weight *w(x*_*i*_, *x*_*s, j*_*)* is computed as:

(2) $$w(x_{i},x_{s,j})=\{\begin{array}{c} \begin{array}{cc} \exp^{\frac{-{\left\|P(x_{i})-P(x_{s,j})\right\|}_{2}^{2}}{h^{2}}} & \begin{array}{cc} if & ss>th \end{array} \end{array}\\ \begin{array}{cc} 0 & \begin{array}{cc} & otherwise \end{array} \end{array} \end{array},$$

where ||.||_2_ is the L2-norm computed between each intensity of the elements of the patches *P(x*_*i*_*)* and *P(x*_*s, j*_*)*. If the structure similarity *ss* between the patches is less than *th*, the weight is not computed and is set directly to zero.

Finally, by considering the labels *y* defined in {0, 1}, the final label *L(x*_*i*_*)* is computed as:

(3) $$L(x_{i})=\{\begin{array}{c} \begin{array}{cc} 1 & v(x_{i})>0.5 \end{array}\\ \begin{array}{cc} 0 & v(x_{i})<0.5 \end{array} \end{array}$$

In the updated method in volBrain pipeline we have modified the voting scheme in several ways. First, we make use of the locality principle assuming that samples closer in the space are likely to be also similar on their label. Therefore, we redefine the similarity weight to take into account not only intensity similarity but also patch spatial proximity:

(4) $$w(x_{i},x_{s,j})=\{\exp^{\frac{{\left|\left\|xi-xj\right\|\right|}^{2}}{\sigma \begin{array}{c} 2\\ d \end{array}}}\;\begin{array}{c} \exp^{\frac{-{\left\|P(x_{i})-P(x_{s,j})\right\|}_{2}^{2}}{h^{2}}}\\ \begin{array}{cc} 0 & \begin{array}{cc} & otherwise \end{array} \end{array} \end{array}\;\begin{array}{cc} if & ss>th \end{array}\;$$

where *x*_*i*_ and *x*_*j*_ are the coordinates of patch centers and σ_*d*_ is normalization constant (σ_*d*_ = √2 mm was found to be optimal in our experiments). As can be noticed, this approach shares some similarities to the bilateral filter proposed by Tomasi and Manduchi (1998) for image denoising.

Finally, a comment about *h* parameter of Equation (4) has to done since it plays a major role in the weight computation process. In Coupé et al. (2011) this value was set to:

(5) $$h^{2}(x_{i})=\lambda \begin{array}{cc} \underset{x_{s,j}}{\min} & {\left\|P(x_{i})-P(x_{j,s})\right\|}_{2}^{2} \end{array}+\varepsilon$$

where ε is a small constant to ensure numerical stability in case the patch under consideration is contained in the library. In Coupé et al. (2011) λ was set to 1 but we found experimentally that a value of 0.15 produced better results in the proposed method probably due to the improved intensity normalization.

On the other hand, Equation (1) was modified to allow multiple *M* tags instead of a single binary decision.

(6) $$v(x_{i},k)=\frac{\sum_{s=1}^{N}\sum_{j\in V_{i}}w(x_{i},x_{s,j}).\delta (k,y_{s,j})}{\sum_{k=0}^{M}\sum_{s=1}^{N}\sum_{j\in V_{i}}w(x_{i},x_{s,j})}$$

where δ is the Kroenecker's delta function and *k* = [0, *M*]. Finally, for each voxel, the most voted label is selected as its label:

(7) $$L(x_{i})=\arg\max_{k}v(x_{i},k)$$

We noted that since the classical non-local label fusion works on a voxel-wise manner there is a lack of regularization on the final labels which is common property of the anatomical structures. To intrinsically provide some degree of label regularity we used a block-wise vote scheme similar to the one proposed by Coupé et al. (2008) for denoising and applied to segmentation in Rousseau et al. (2011). So defined the new block-wise votes are computed as follows:

(8) $$v(B(x_{i}),k)=\frac{\sum_{s=1}^{N}\sum_{j\in V_{i}}w(x_{i},x_{s,j}).\delta (k,B(y_{s,j})}{\sum_{k=0}^{M}\sum_{s=1}^{N}\sum_{j\in V_{i}}w(x_{i},x_{s,j})}$$

where *B*(*x*_*i*_) is a 3D region which is labeled at the same time. Finally, the vote count *v*(*x*_*i*_, *k*) for the voxel *x*_*i*_ is obtained in an overcomplete manner by summing over all blocks containing *x*_*i*_, i.e.,

(9) $$v\left(x_{i},k\right)=\sum_{x_{i}\in B(z)}{\left[v(B\left(z\right),k)\right]}_{i}$$

where [*v*(B(*z*),*k*)]_*i*_ refers to the element corresponding to *x*_*i*_ in the block *v*(*B*(*z*),*k*) and the label *L*(*x*_*i*_) is decided as in Equation (7).

As shown in Coupé et al. (2011), different brain structures may require different parameter settings to accurately capture their properties. Basically this normally requires changing the patch size in the similarity estimation (Equation 4) depending on each structure size. An automatic way to perform this multiscale process is to compute the patch similarities with different patch sizes and then perform a late fusion of their contributions prior estimating final label (Equation 8).

(10) $$v(x_{i},k)=0.5v_{1}(x_{i},k)+0.5v_{2}(x_{i},k)\;\forall x_{i}\forall k$$

Here *v*_1_ and *v*_2_ represent the weights at each position and label for patch sizes *P*_1_ and *P*_2_. Final map *v* is simply estimated as the mean of both contributions. Note that although an early aggregation could be done (that is combining *P*_1_ and *P*_2_ in a single similarity measure) this not necessarily the best strategy as we confirmed experimentally.

As in Coupé et al. (2011) an exhaustive search for optimal parameter settings was performed. First, we studied the performance of the proposed method in function of the number *N* of selected cases from the library. As expected, increasing the number of selected training subjects increased the accuracy of the segmentation. By using *N* = 25, we found an optimal setting between accuracy and computational burden, this is in good agreement with the previous version of the method where the plateau was met around 20. To further increase the quality of the segmentation a larger library must be used. We also studied the impact of the 3D patch size and the 3D neighborhood on segmentation accuracy. Optimal setting was found to be patch size P1 set to 3 × 3 × 3 voxels and patch size P2 set to 5 × 5 × 5. Finally, the search volume was set to 9 × 9 × 9 voxels which was found a good compromise between quality and computational burden.

### The volBrain online system

Most of the developed pipelines for MR image analysis are packages that need to be downloaded, installed and configured. Installation step can be complicated and thus may require an experimented person not always available in a research laboratory or clinical context. In addition, the users have to be trained to use the software and computational resources have to be allocated to run it. These requirements can make complex the use of these packages, especially the most recent and sophisticated ones since they usually require high hardware requirements. Furthermore, multiplatform versions and support has to be deployed to the community of users.

We have tried to overcome all these problems by deploying our proposed pipeline through a web interface (http://volbrain.upv.es) providing not only access to the software pipeline but also sharing the computational resources of our institution. Thus, using the volBrain pipeline does not require any installation, configuration or training. The volBrain volumetric analysis system works remotely through a web interface using a SaaS (Software as a Service) model to automatically provide a report containing volumetric information from any submitted case. The volBrain interface is supported by an XAMPP web server on a Windows 7 system which has been developed in AJAX (HTML + Javascript + PHP) + MySQL. The segmentation pipeline is executed on dedicated cluster running Windows Server 2012 R2 Datacenter using a Matlab compiled version of our pipeline (the proposed method was fully implemented in MATLAB© 7.8.0 by using MEX code). The system runs on a cluster consisting of seven machines DELL PowerEdge R720 with two processors Intel Xeon E5-2620 (12 cores total per machine) and 64 GB of RAM each one. The system has been designed to deal with up to 14 concurrent volBrain jobs and has theoretical limit of 1200 processed cases per day.

**Figure 2:**
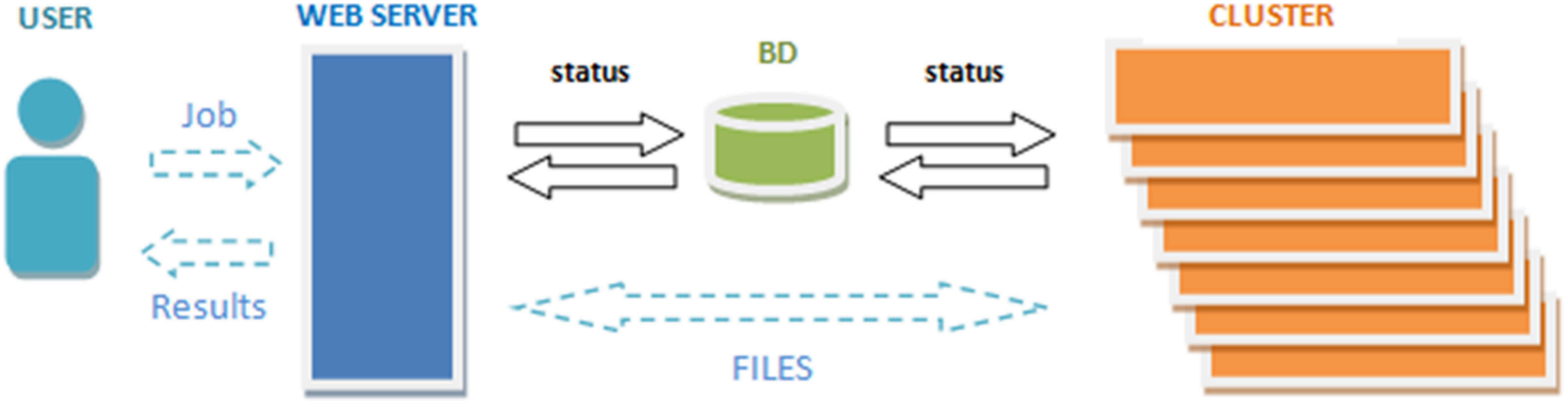
**The volBrain system processing scheme**.

**Figure 3:**
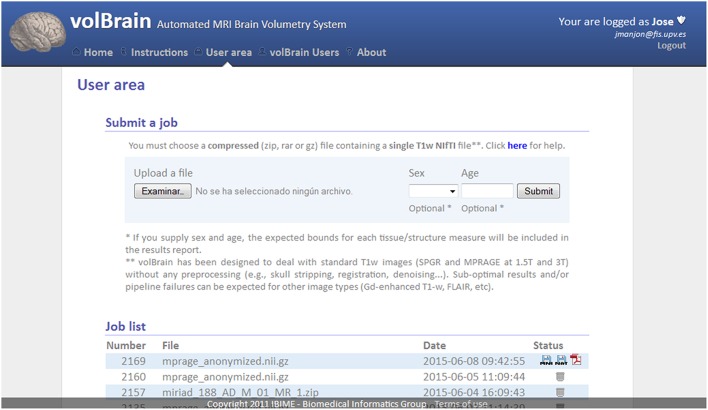
**Capture of volBrain website user area**. Here the user can submit a new case and download the results of previous cases.

To get access to the system any user is asked to register by providing some personal information such as the email, name and the institution name he/she belongs. The terms of use the system are showed in the web page making special remark about the use of the data for research purposes only. The web server (see Figure 2) accepts requests (jobs) from users who submit a single anonymized compressed MRI T1w Nifti file through a web interface (see Figure 3). The web-server also dispatches the computational load among the seven available machines. This job dispatching is done by using a queuing system consisting of a main queue (FIFO) for the incoming jobs and a process queue (FIFO) for each cluster machine.

The web-cluster communication is bidirectional and it is implemented by several PHP daemons running on the web server and the cluster machines. Jobs are assigned to a machine using database entries that are periodically consulted by the daemons on the web server and the cluster. The cluster also notifies its current status by updating these entries. The files from the web server are transferred to the processing machines by secured FTP and the results from the processing machines are also sent to the web server which storages the results and send the corresponding reports to the users (see Figure 2).

The output produced by the volBrain pipeline consists in a pdf and csv files sent to the user by email. These files summarize the volumes and asymmetry ratios estimated from their data. If the user provides the sex and age of the submitted subject, population-based normal volumes and asymmetry bounds for all structures are added for reference purposes. This normality bounds were automatically estimated from the IXI dataset which contains almost 600 normal subjects covering most of adult lifespan.

**Figure 4:**
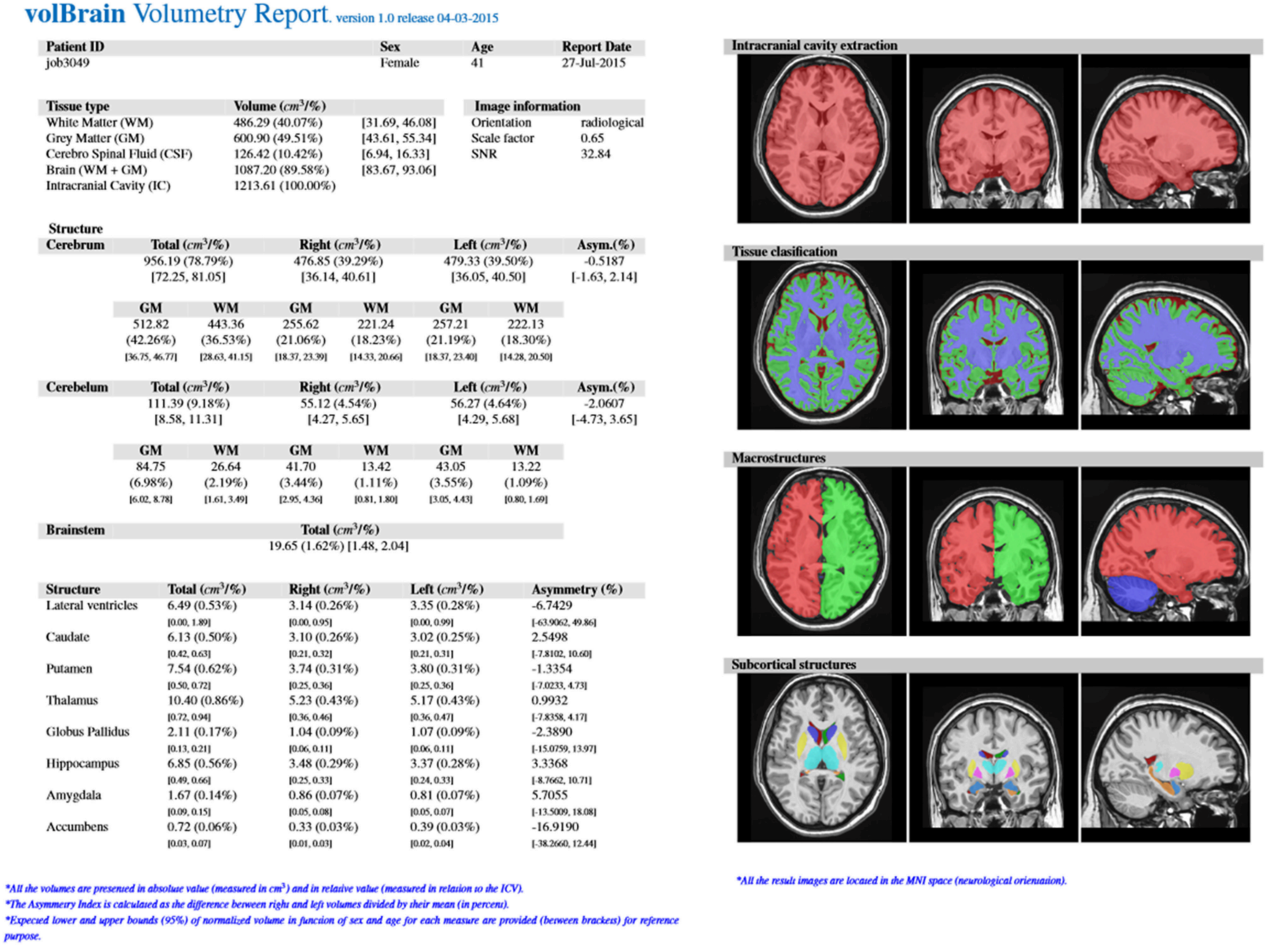
**Example of volBrain pdf report**.

Furthermore, the user can access to its user area through volBrain website (see Figure 3) to download the resulting Nifti files containing the segmentations at different scales (both in native or MNI space). Figure 4 shows an example of a volumetry report produced by volBrain (note that a screenshot of the results is included for quality control purposes).

## Experiments and results

In this section some experimental results are shown to highlight the accuracy and reproducibility of the proposed pipeline.

Since volBrain provides results at different scales both accuracy and reproducibility at each scale will be commented. Specifically, we will comment the results for intracranial cavity extraction (NICE), Macrostructure segmentation (NABS) and subcortical structure segmentation. Note that the tissue classification is not included in this evaluation since it is based on our particular way to compute PVCs. Therefore, there is no a direct comparison with methods like SPM or VBM.

NICE results were already presented recently in its corresponding paper (Manjón et al., 2014). To summarize, NICE was compared with BEaST and VBM8 and it was found to be significantly better (it obtained the best DICE coefficient (0.9911) compared to BEAST (0.9880) and VBM8 (0.9762)). Moreover, an independent test was also performed using the SVE website (see https://www.nitrc.org/projects/sve/) were NICE ranked first (NICE was still first at the time of writing this paper). Regarding the reproducibility, NICE was found to be the most reproducible method followed by VBM8 and finally BEAST. For further details we recommend the reader the original NICE paper (Manjón et al., 2014).

NABS method was also recently evaluated in its corresponding paper. Summarizing, NABS was compared with ADISC (Zhao et al., 2010) and it obtained a significantly better DICE coefficient (0.9962) compared to ADISC (0.9868). NABS method was also compared to ADISC method using an application consisting on estimating brain asymmetries on AD cases. We showed that NABS method was able to better predict the patient status. Again, further details can be found in the original paper (Romero et al., 2015).

Finally, experiments to measure both accuracy and reproducibility of the proposed subcortical segmentation method were performed and comparison with *state-of-the-art* related approaches are presented.

### Accuracy

A leave-two-out procedure was performed for the 50 subjects of the library (i.e., excluding the case to be segmented and its mirrored version). Dice's kappa (Zijdenbos et al., 1994) was then computed by comparing the manual segmentations with the segmentations obtained with our method. The proposed method was also compared with two publically available software packages for subcortical brain structures labeling (Freesurfer, Fischl et al., 2002) and FSL-FIRST (Patenaude et al., 2011). Both methods were run on the CBRAIN interface (http://mcin-cnim.ca/neuroimagingtechnologies/cbrain/) with their default parameters (Tarek et al., 2014).

**Figure 5:**
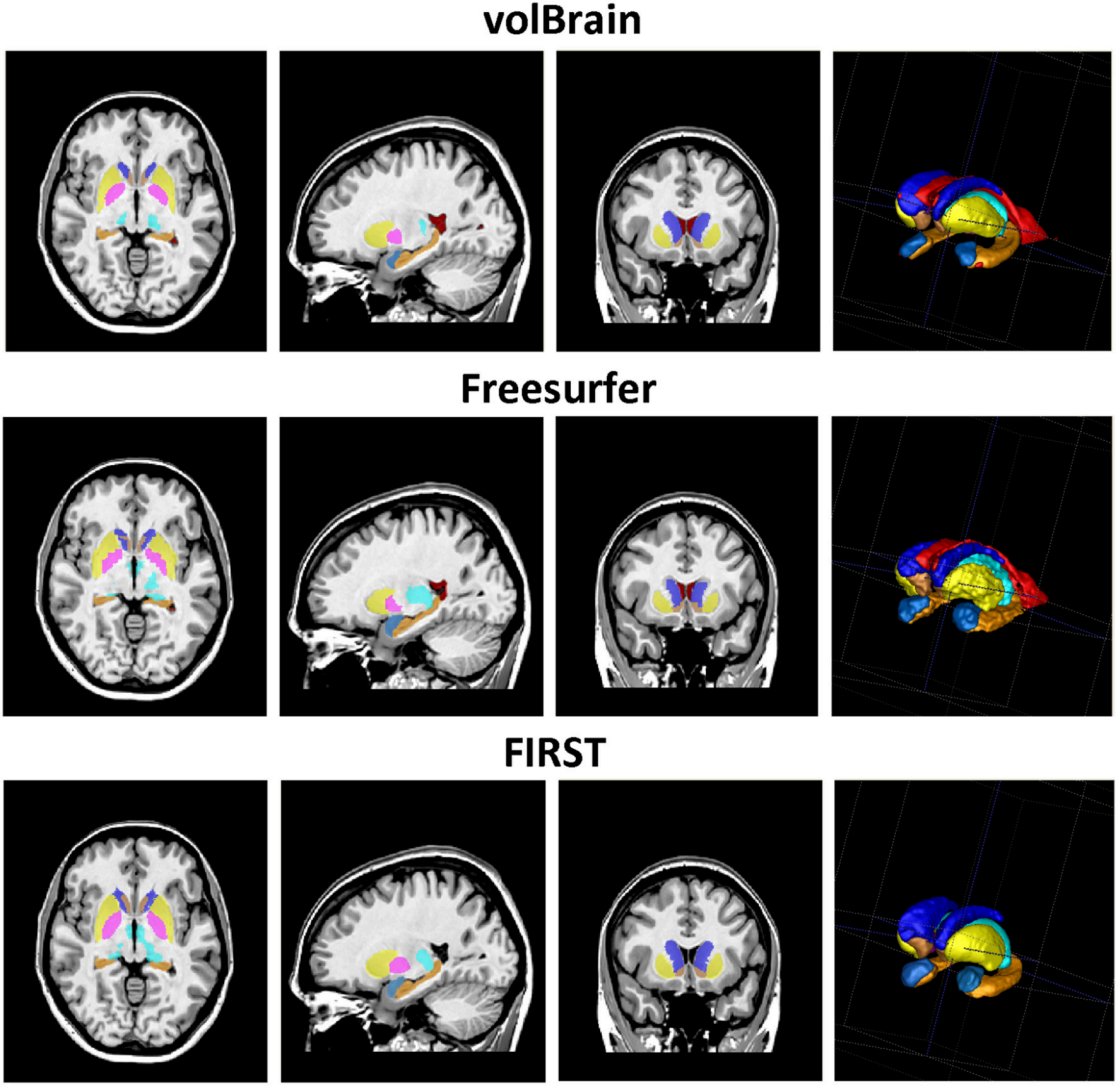
**Visual example of the segmentation results (Axial, sagittal, and coronal views and 3D representation of segmented subcortical structures)**. First row: volBrain results. Second row, Freesurfer results; Third row, FIRST results. Note that FIRST output does not include lateral ventricles.

As can be noted on Table 1, volBrain obtained the best DICE coefficients for all the considered structures. Moreover, the improvement was statistically significant for all the structures and for the two methods compared. VolBrain obtained an average dice coefficient (without including lateral ventricles) of 0.9136 while Freesurfer obtained 0.7327 and FIRST 0.7706. A visual comparison of one sample case is showed Figure 5 were the labeling of the three different methods are presented with 3D representation (note that FIRST does not segment lateral ventricles and therefore they are not included in the comparative). On one hand, Freesurfer method produced a rough segmentation of the different structures with significant errors. On the other hand, FIRST performed better and produced smooth surfaces on the different structures. However, FIRST method seems to over segment most of the structures.

**Table 1:** **Mean Dice coefficient of the different subcortical structures over the 50 cases of template library**.

**Structure**	**volBrain**	**Freesurfer**	**FIRST**
Lat. Ventricles	0.9836 ± 0.0111^*^	0.8315 ± 0.0589	—
Caudate	0.9427 ± 0.0196^*^^†^	0.8195 ± 0.0418	0.8366 ± 0.0706
Putamen	0.9442 ± 0.0226 ^*^^†^	0.8162 ± 0.0396	0.8775 ± 0.0192
Thalamus	0.9476 ± 0.0196 ^*^^†^	0.8157 ± 0.0247	0.8144 ± 0.0322
Pallidum	0.8914 ± 0.0403 ^*^^†^	0.7454 ± 0.0906	0.7851 ± 0.0575
Hippocampus	0.9533 ± 0.0092 ^*^^†^	0.7886 ± 0.0254	0.8429 ± 0.0303
Amygdala	0.8795 ± 0.0559 ^*^^†^	0.5844 ± 0.1092	0.5895 ± 0.0962
Accumbens	0.8362 ± 0.0572 ^*^^†^	0.5589 ± 0.0697	0.6483 ± 0.0916
Average	0.9224 ± 0.0570^*^^†^	0.7546 ± 0.1198	—
Average	0.9136 ± 0.0555 ^*^^†^	0.7327 ± 0.1132	0.7706 ± 0.1087

*Average represents the average dice without including lateral ventricles. Best results are in bold*.

* *Indicates statistically significant differences between volBrain and Freesurfer (p < 0.05)*.

† *Indicates statistically significant differences between volBrain and FIRST*.

**Figure 6:**
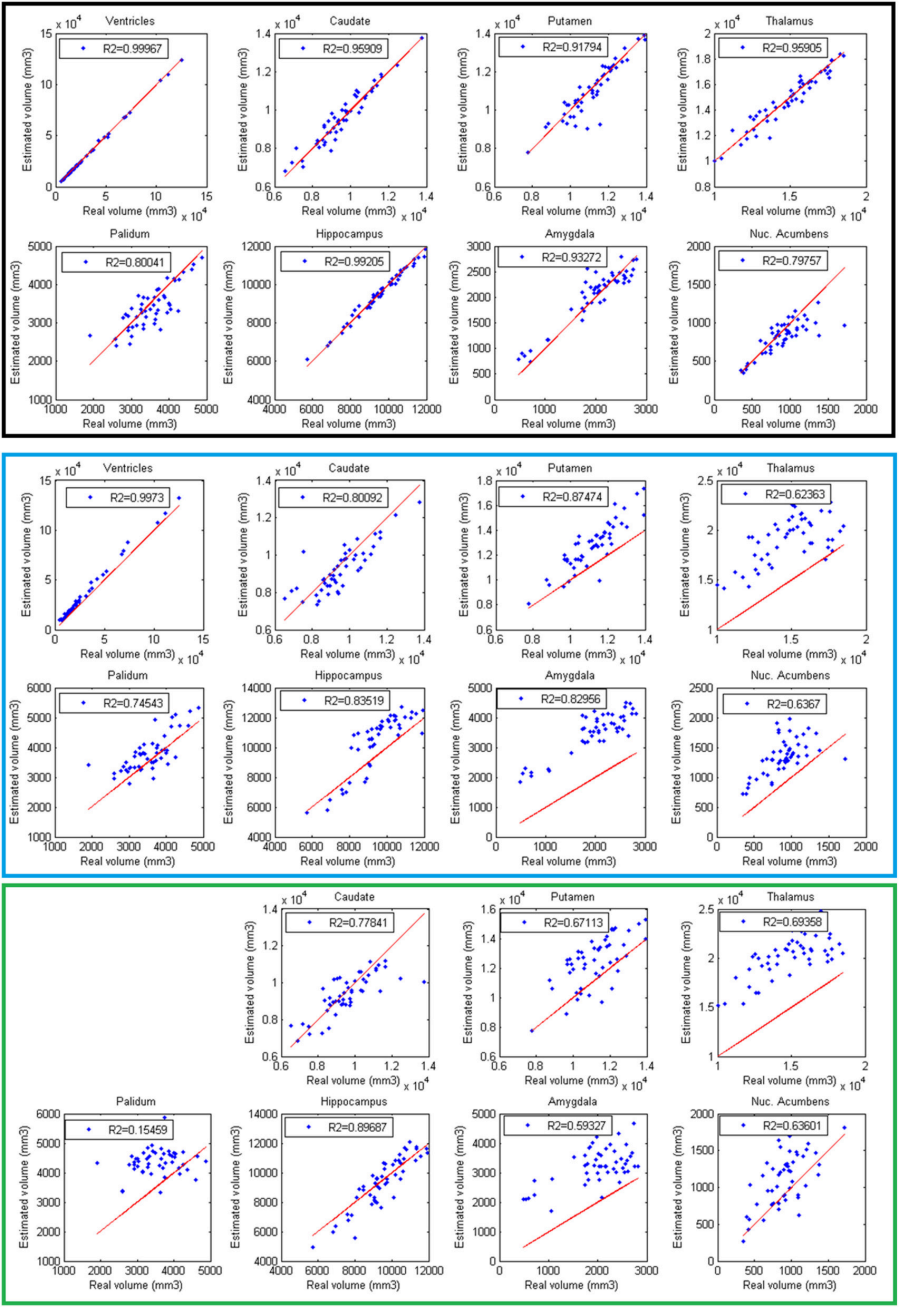
**Correlation between volumes obtained with volBrain (Black, first 2 rows), Freesurfer (Blue, 3 and 4 rows), and FIRST (Green, last 2 rows) and volumes obtained with manual segmentations considered as the gold standard**. Red lines represent identity.

Finally, volumes obtained with the automatic methods were compared with volumes obtained with manual segmentations considered as the gold standard. In Figure 6, the results of the different methods are displayed. The proposed method produced consistent volumes showing higher correlation with volumes obtained by manual segmentations. In addition, Freesurfer and FIRST showed a greater dispersion on the measures. As can be seen on Figure 6, FIRST severely overestimate the volumes of most of the structures.

We are aware that the presented volume and accuracy results are slightly biased in favor to volBrain due to the use of the same label definition for training and validation. However, minimal differences on label definition where used compared to FIRST or Freesurfer labels with the exception of lateral ventricles (we did not include choroid plexus) and hippocampus (we used EADC protocol). Besides, it has been recently shown by using a joint DTI MRI analysis (Næss-Schmidt et al., 2016) that Freesurfer and FIRST overestimate most of subcortical structures that matches with our findings. In summary, the large differences found among the compared methods provide evidences of the high quality of the proposed pipeline. A particular mention has to be done for hippocampus segmentation. Since we used the harmonized EADC protocol (Frisoni et al., 2015), we can conclude that volBrain is the most accurate method to segment hippocampus according to this protocol. This is especially important given that fact that EADC protocol is the new consensus protocol for hippocampus analysis for Alzheimer's disease.

### Reproducibility

A very important feature for a measurement method is its reproducibility. To measure the reproducibility of the different compared methods, we used a reproducibility dataset of the brain segmentation testing protocol website (https://sites.google.com/site/brainseg/). This dataset consists of a test-retest set of 20 subjects scanned twice in the same scanner and with the same sequence (SSS).

To measure the reproducibility of the two repeated sets we used the Percent Volume Difference (PVD) and the Percent Volume Overlap (PVO) (Morey et al., 2010) defined as follows:

(11) $$\begin{array}{l} PVD(C_{1},C_{2})=100\frac{2\left|{\left\|C_{1}\right\|}_{0}-{\left\|C_{2}\right\|}_{0}\right|}{{\left\|C_{1}\right\|}_{0}+{\left\|C_{2}\right\|}_{0}}\\ PVO(C_{1},C_{2})=100\frac{2{\left\|\left|C_{1}-C_{2}\right|\right\|}_{0}}{{\left\|C_{1}\right\|}_{0}+{\left\|C_{2}\right\|}_{0}} \end{array}$$

where *C*_1_ and *C*_2_ represent the segmentations 1 and 2 respectively. Note that the reference to compute the percentage is set to the mean of both segmentations to avoid any order influence.

#### SSS dataset

This reproducibility dataset consist in a subset of the OASIS (www.oasis-brains.org) dataset consisting in 20 subjects (age = 23.4 ± 4.0 years, 8 females) who were scanned using the same pulse sequence two times (1.5 T Siemens Vision scanner, *TR* = 9.7 ms, *TE* = 4 ms, *TI* = 20 ms, flip angle = 10°, slice thickness = 1.25 mm, matrix size = 256 × 256, voxel dimensions = 1 × 1 × 1.25 mm3 resliced to 1 × 1 × 1 mm^3^, averages = 1).

The three compared methods were run on this dataset but the comparison was done only on a subset of 18 subjects since FIRST did not produce valid results for two of the 20 cases. Since PVD and PVO measures do not distribute normally we represent the results using the median and the interquartile interval and we used the Wilcoxon rank test to measure the statistical significance of the differences between methods. Results of this comparison are summarized on Tables 2, 3. As can be noted, volBrain was more reproducible in general compared to Freesurfer and FIRST (although the differences were not statistically significant overall).

**Table 2:** **Median of PVD**.

**Structure**	**volBrain**	**Freesurfer**	**FIRST**
Lat. Ventricles	**4.95 [7.42]**	5.06 [4.85]	—
Caudate	**0.53 [1.22]**	1.39 [2.47]	1.19 [1.20]
Putamen	0.69 [1.76]^*^	2.23 [2.90]	1.20 [1.23]
Thalamus	**0.82 [1.04]**	0.93 [0.66]	0.98 [1.67]
Pallidum	1.60 [1.92]	3.18 [3.72]	**0.92 [0.96]^†^**
Hippocampus	**1.41 [2.97]**	1.73 [1.74]	2.15 [3.20]
Amygdala	**3.38 [2.06]**	4.13 [5.23]	3.94 [3.63]
Accumbens	**2.65 [22.81]**	2.68 [2.48]	4.26 [6.92]
Overall	**1.59**	2.33	2.09

*volBrain was compared to the other two methods (corresponding interquartile interval is shown in brackets). Best results are in bold*.

* *Indicates statistically significant differences between volBrain and Freesurfer (p < 0.05)*.

† *Indicates statistically significant differences between volBrain and FIRST. Overall represents the mean PVD of all structures excluding lateral ventricles to enable direct global comparison of the three methods*.

**Table 3:** **Median of PVO**.

**Structure**	**volBrain**	**Freesurfer**	**FIRST**
Lat. Ventricles	**94.09 [4.71]**	90.79 [4.33]	—
Caudate	96.96 [3.06]^*^	91.65 [1.85]	96.62 [1.12]
Putamen	97.21 [1.34]^*^	92.36 [1.50]	**97.37 [0.76]**
Thalamus	97.38 [1.24]^*^	93.58 [1.38]	**98.22 [1.18]^†^**
Pallidum	95.65 [1.19]^*^	86.29 [5.95]	**96.92 [1.32]^†^**
Hippocampus	96.19 [1.88]^*^^†^	90.90 [1.08]	93.80 [2.04]
Amygdala	93.25 [2.74]^*^^†^	87.78 [1.43]	91.37 [2.63]
Accumbens	93.10 [13.01]^*^	84.55 [3.46]	91.91 [2.55]
Overall	**95.68**	89.59	95.17

*volBrain was compared to the other two methods (corresponding interquartile interval is shown in brackets). Best results are in bold*.

* *Indicates statistically significant differences between volBrain and Freesurfer (p < 0.05)*.

† *Indicates statistically significant differences between volBrain and FIRST. Overall represents the mean PVO of all structures excluding lateral ventricles to enable direct global comparison of the three methods*.

Regarding to the volume estimation, volBrain was found to be significantly more reproducible than Freesurfer for putamen (*p* < 0.05) while FIRST was significantly better than volBrain and Freesurfer for the pallidum. In relation to the segmentation masks reproducibility, volBrain was found to be most reproducible method overall. Especially, volBrain was significantly more reproducible than Freesurfer for all structures with the exception of lateral ventricles. Compared to FIRST, volBrain was found more reproducible for hippocampus and amygdala while FIRST was more reproducible for thalamus and pallidum.

### Computational time

The proposed method takes around 12 min in average to complete the whole pipeline, this included 30 s for denoising, 30 s for inhomogeneity correction, 2 min to perform registration into MNI space, 3 min for SPM inhomogeneity correction, 5 s for intensity normalization, 2 min to do brain extraction, 5 s to perform tissue classification, 2 min for NABS and 3 min for structures labeling. Freesurfer normally takes around 15 h to perform the complete analysis (which includes also surface extraction). FIRST running time is approximately 10 min (only for the subcortical structure segmentation).

## Discussion

We proposed a novel pipeline that is able to automatically segment the brain at different scales in a robust and efficient manner using a library of expert segmentations used as priors and an enhanced version of our patch-based label fusion scheme. We have shown that the proposed pipeline is able to provide *state-of-the-art* results at different levels (intracranial cavity, brain macro-structures and subcortical structures) in a very efficient manner.

The proposed pipeline was compared with two well-established software packages (Freesurfer and FIRST) for subcortical structure segmentation. The volBrain pipeline was found to significantly improve the accuracy (according to the used protocol) compared to both methods. We should also remark that volBrain platform is one of the first few platforms to provide hippocampus segmentation based on EADC protocol that will be the reference definition on AD in the next years. Regarding to the reproducibility, volBrain was also found to be the more reproducible than Freesurfer and FIRST overall for both volume and shape estimation. This is an important issue since the higher the reproducibility the higher the chances to detect subtle variations induced by the disease. In addition, we found that segmentation masks obtained with FIRST were more accurate and more reproducible than Freesurfer ones. The results on volume reproducibility between Freesurfer and FIRST were less obvious since they were structure dependent. However, it has to be noted that FIRST failed for 2 cases of 20 (i.e., 10% of failure rate) while both volBrain and Freesurfer worked for all the 20 cases. The high failure rate of FIRST can limit it use in clinical context (Kempton et al., 2011). Finally, volBrain pipeline was also found more computationally efficient than Freesurfer since it takes few minutes to produce the results compared to several hours in the case of Freesurfer (we have to note that Freesurfer provides full brain segmentation and cortical thickness in this time) and similar than FISRT (only for subcortical segmentation without lateral ventricles).

Moreover, an online web-based platform has been deployed that gives access to the whole scientific community not only to the software pipeline but also to our own computational resources (we limit the number of cases that a user can submit daily to 10 in order to share our system to the higher possible number of users). Although, our computational resources are limited, the efficiency of the proposed pipeline allows processing around 1200 subjects per day. At the day of writing this paper, the system had already more than 550 registered users from 284 different institutions from all around the world and has automatically processed more than 10,000 subjects during its first year with a failure rate lower than the 2%. The peak activity of our system was 348 cases in 1 day without major problems. However, given the modularity and scalability of our system, we can easily expand the number of computational nodes by adding more computers locally or by using external cloud-based processing nodes. From the user's perspective, we have been informed that to upload a case to the system takes from few seconds to 1 min (for very slow connections or big size files). Besides, the lack of parameter tuning and the easiness of use have been highlighted by several users as a really valuable feature of the system.

## Conclusion

In this paper, we present a novel pipeline based on our state-of-the-art non-local label fusion technology to segment brain anatomical structures at different scales. The proposed pipeline has been compared with state-of-the-art related packages showing competitive results in term of accuracy, reproducibility and computational time. Besides, we hope that the online nature of the proposed pipeline will facilitate the access of any user around the world to the proposed system making their MRI data analysis simpler and more efficient. We plan to extend the volBrain capabilities in the near future to segment all the brain structures including cortical areas and to add regional cortical thickness measurements in the final report.

## Author contributions

All authors listed, have made substantial, direct and intellectual contribution to the work, and approved it for publication.

### Conflict of interest statement

The authors declare that the research was conducted in the absence of any commercial or financial relationships that could be construed as a potential conflict of interest.

## References

[B1] Ahmed M. N. Yamany S. M. Nevin M. Farag A. A. Moriarty T. (2002). A modified fuzzy C-Means algorithm for bias field estimation and segmentation of MRI data. IEEE Trans. Med. Imaging 21, 193. 10.1109/42.996338 11989844

[B2] Ashburner J. Friston K. J. (2005). Unified segmentation. Neuroimage 26, 839–851. 10.1016/j.neuroimage.2005.02.018 15955494

[B3] Avants B. B. Tustison N. Song G. (2009). Advanced normalization tools (ANTS). Insight J. 2, 1–35.

[B4] Barnes J. Foster J. Boyes R. G. Pepple T. Moore E. K. Schott J. M.. (2008). A comparison of methods for the automated calculation of volumes and atrophy rates in the hippocampus. Neuroimage 40, 1655–1671. 10.1016/j.neuroimage.2008.01.012 18353687

[B5] Brummer M. (1991). Hough transform detection of the longitudinal fissure in tomographic head images. IEEE Trans. Med. Imaging 10, 74–81. 10.1109/42.75613 18222802

[B6] Buades A. Coll B. Morel J. M. (2005). A non local algorithm for image denoising, in IEEE International Conference on Computer Vision and Pattern Recognition, CVPR 2005, Vol. 2 (San Diego, CA), 60–65.

[B7] Chupin M. Mukuna-Bantumbakulu A. R. Hasboun D. Bardinet E. Baillet S. Kinkingnéhun S.. (2007). Anatomically constrained region deformation for the automated segmentation of the hippocampus and the amygdala: method and validation on controls and patients with Alzheimer's disease. Neuroimage 34, 996–1019. 10.1016/j.neuroimage.2006.10.035 17178234

[B8] Collins D. L. Holmes C. J. Peters T. M. Evans A. C. (1995). Automatic 3-D model-based neuroanatomical segmentation. Hum. Brain Mapp. 3, 190–208. 10.1002/hbm.460030304

[B9] Collins D. L. Pruessner J. C. (2010). Towards accurate, automatic segmentation of the hippocampus and amygdala from MRI by augmenting ANIMAL with a template library and label fusion. Neuroimage 52, 1355–1366. 10.1016/j.neuroimage.2010.04.193 20441794

[B10] Coupé P. Eskildsen S. F. Manjón J. V. Fonov V. Collins D. L. ADNI. (2012). Simultaneous segmentation and grading of anatomical structures for patient's classification: application to Alzheimer's disease. Neuroimage 59, 3736–3747. 10.1016/j.neuroimage.2011.10.080 22094645

[B11] Coupé P. Manjón J. V. Fonov V. Pruessner J. Robles M. Collins D. L. (2011). Patch-based segmentation using expert priors: application to hippocampus and ventricle segmentation. Neuroimage 54, 940–954. 10.1016/j.neuroimage.2010.09.018 20851199

[B12] Coupé P. Yger P. Prima S. Hellier P. Kervrann C. Barillot C. (2008). An optimized blockwise nonlocal means denoising filter for 3-D magnetic resonance images. IEEE Trans. Med. Imaging 27, 425–441. 10.1109/TMI.2007.906087 18390341 PMC2881565

[B13] Eskildsen S. F. Coupé P. Leung K. K. Fonov V. Manjón J. V. Guizard N.. (2012). BEaST: Brain Extraction based on nonlocal Segmentation Technique. Neuroimage 59, 2362–2373. 10.1016/j.neuroimage.2011.09.012 21945694

[B14] Fischl B. Salat D. H. Busa E. Albert M. Dieterich M. Haselgrove C.. (2002). Whole brain segmentation: automated labeling of neuroanatomical structures in the human brain. Neuron 33, 341–355. 10.1016/S0896-6273(02)00569-X 11832223

[B15] Frisoni G. B. Jack C. R. (2011). Harmonization of magnetic resonance-based manual hippocampal segmentation: a mandatory step for wide clinical use. Alzheimer's Dement. 7, 171–174. 10.1016/j.jalz.2010.06.007 21414554

[B16] Frisoni G. B. Jack C. R. Jr. Bocchetta M. Bauer C. Frederiksen K. S. Liu Y.. (2015). The EADC-ADNI Harmonized Protocol for manual hippocampal segmentation on magnetic resonance: evidence of validity. Alzheimers Dement. 11, 111–125. 10.1016/j.jalz.2014.05.1756 25267715 PMC4422168

[B17] Gousias I. S. Rueckert D. Heckemann R. A. Dyet L. E. Boardman J. P. Edwards A. D.. (2008). Automatic segmentation of brain MRIs of 2-year-olds into 83 regions of interest. Neuroimage 40, 672–684. 10.1016/j.neuroimage.2007.11.034 18234511

[B18] Hata Y. Kobashi S. Hirano S. Kitagaki H. Mori E. (2000). Automated segmentation of human brain MR images aided by fuzzy information granulation and fuzzy inference. IEEE Trans. Sys. Man Cybernet. C Appli. Rev. 30, 381–395. 10.1109/5326.885120

[B19] Heckemann R. A. Hajnal J. V. Aljabar P. Rueckert D. Hammers A. (2006). Automatic anatomical brain MRI segmentation combining label propagation and decision fusion. Neuroimage 33, 115–126. 10.1016/j.neuroimage.2006.05.061 16860573

[B20] Ibáñez L. Schroeder W. Ng L. Cates J. Consortium T. I. S. Hamming R. (2003). The ITK Software Guide. Clifton Park, NY: Kitware.

[B21] Jenkinson M. Beckmann C. F. Behrens T. E. Woolrich M. W. Smith S. M. (2012). FSL. Neuroimage 62, 782–790. 10.1016/j.neuroimage.2011.09.015 21979382

[B22] Kempton M. J. Underwood T. S. Brunton S. Stylios F. Schmechtig A. Ettinger U.. (2011). A comprehensive testing protocol for MRI neuroanatomical segmentation techniques: evaluation of a novel lateral ventricle segmentation method. Neuroimage 58, 1051–1059. 10.1016/j.neuroimage.2011.06.080 21835253 PMC3551263

[B23] Larsson J. (2001). Imaging Vision: Functional Mapping of Intermediate Visual Processes in Man. Ph.D. thesis, Karolinska Institutet, Stockholm.

[B24] Leung K. K. Barnes J. Modat M. Ridgway G. R. Bartlett J. W. Fox N. C.. (2011). Brain MAPS: an automated, accurate and robust brain extraction technique using a template library. Neuroimage 55, 1091–1108. 10.1016/j.neuroimage.2010.12.067 21195780 PMC3554789

[B25] Lötjönen J. M. Wolz R. Koikkalainen J. R. Thurfjell L. Waldemar G. Soininen H.. (2010). Fast and robust multi-atlas segmentation of brain magnetic resonance images. Neuroimage 49, 2352–2365. 10.1016/j.neuroimage.2009.10.026 19857578

[B26] Maes F. Van Leemput K. DeLisi L. Vandermeulen D. Suetens P. (1999). Quantification of cerebral grey and white matter asymmetry from MRI. Lecture Notes Comput. Sci. 1679, 348–357. 10.1007/10704282_38

[B27] Mangin J.-F. Rivière D. Cachia A. Duchesnay E. Cointepas Y. Papadopoulos-Orfanos D.. (2004). A framework to study the cortical folding patterns. Neuroimage 23, 129–138. 10.1016/j.neuroimage.2004.07.019 15501082

[B28] Manjón J. V. Coupé P. Martí-Bonmatí L. Collins D. L. Robles M. (2010b). Adaptive non-local means denoising of MR images with spatially varying noise levels. J. Magnet. Reson. Imaging 31, 192–203. 10.1002/jmri.22003 20027588

[B29] Manjón J. V. Eskildsen S. F. Coupé P. Romero J. E. Collins D. L. Robles M. (2014). Non-local intracranial cavity extraction. Int. J. Biomed. Imaging 2014:820205. 10.1155/2014/820205 25328511 PMC4195262

[B30] Manjón J. V. Tohka J. García-Martí G. Carbonell-Caballero J. Lull J. J. Martí-Bonmatí L.. (2008). Robust MRI brain tissue parameter estimation by multistage outlier rejection. Magn. Reson. Med. 59, 866–873. 10.1002/mrm.21521 18383286

[B31] Manjón J. V. Tohka J. Robles M. (2010a). Improved estimates of partial volume coefficients from noisy Brain MRI using spatial context. Neuroimage 53, 480–490. 10.1016/j.neuroimage.2010.06.046 20600978

[B32] Morey R. A. Selgrade E. S. Wagner H. R. II Huettel S. A. Wang L. McCarthy G. (2010). Scan-rescan reliability of subcortical brain volumes derived from automated segmentation. Hum. Brain Mapp. 31, 1751–1762. 10.1002/hbm.20973 20162602 PMC3782252

[B33] Næss-Schmidt E. T. Tietze A. Blichera J. U. Petersen M. Mikkelsen I. K. Coupé P.. (2016). Automatic thalamus and hippocampus segmentation from MP2RAGE: comparison of publicly available methods and implications for DTI quantification. IJCARS 2016, 1–13. 10.1007/s11548-016-1433-0 27325140

[B34] Nenadic I. Smesny S. Schlösser R. G. Sauer H. Gaser C. (2010). Auditory hallucinations and brain structure in schizophrenia: a VBM study. Br. J. Psychiatry 196, 412–413. 10.1192/bjp.bp.109.070441 20435970

[B35] Patenaude B. Smith S. M. Kennedy D. Jenkinson M. (2011). A Bayesian model of shape and appearance for subcortical brain segmentation. Neuroimage 56, 907–922. 10.1016/j.neuroimage.2011.02.046 21352927 PMC3417233

[B36] Prima S. Ourselin S. Ayache N. (2002). Computation of the mid-sagittal plane in 3D brain images. IEEE Trans. Med. Imaging 21, 122–138. 10.1109/42.993131 11929100

[B37] Rohlfing T. Brandt R. Menzel R. Maurer C. R. (2004). Evaluation of atlas selection strategies for atlas-based image segmentation with application to confocal microscopy images of bee brains. Neuroimage 21, 1428–1442. 10.1016/j.neuroimage.2003.11.010 15050568

[B38] Romero J. E. Manjón J. V. Tohka J. Coupé P. Robles M. (2015). Non-local automatic Brain hemisphere segmentation. Magnet. Reson. Imaging 33, 474–484. 10.1016/j.mri.2015.02.005 25660644

[B39] Rousseau F. Habas P. A. Studholme C. (2011). A supervised patch-based approach for human brain labeling. IEEE Trans. Med. Imaging 30, 1852–1862. 10.1109/TMI.2011.2156806 21606021 PMC3318921

[B40] Sandor S. Leahy R. (1997). Surface-based labeling of cortical anatomy using a deformable atlas. IEEE Trans. Med. Imaging 16, 41–54. 10.1109/42.552054 9050407

[B41] Shen D. Moffat S. Resnick S. M. Davatzikos C. (2002). Measuring size and shape of the hippocampus in MR images using a deformable shape model. Neuroimage 15, 422–434. 10.1006/nimg.2001.0987 11798276

[B42] Sled J. G. Zijdenbos A. P. Evans A. C. (1998). A nonparametric method for automatic correction of intensity nonuniformity in MRI data. IEEE Trans. Med. Imaging 17, 87–97. 10.1109/42.668698 9617910

[B43] Smith S. M. (2002). Robust automated Brain extraction. Hum. Brain Mapp. 17, 143–155. 10.1002/hbm.10062 12391568 PMC6871816

[B44] Sun C. Sherrah J. (1997). 3D symmetry detection using the extended Gaussian image. IEEE Trans. Pattern Anal. Machine Intellig. 19, 164–168. 10.1109/34.574800

[B45] Tarek S. Rioux P. Rousseau M.-E. Kassis N. Beck N. Adalat R.. (2014). CBRAIN: a web-based, distributed computing platform for collaborative neuroimaging research. Front. Neuroinform. 8:54. 10.3389/fninf.2014.00054 24904400 PMC4033081

[B46] Tohka J. Zijdenbos A. Evans A. C. (2004). Fast and robust parameter estimation for statistical partial volume models in brain MRI. Neuroimage 23, 84–97. 10.1016/j.neuroimage.2004.05.007 15325355

[B47] Tomasi C. Manduchi R. (1998). Bilateral filtering for gray and color images, in Proceedings of the IEEE International Conference on Computer Vision (Bombay), 839–846.

[B48] Tustison N. J. Avants B. B. Cook P. A. Zheng Y. Egan A. Yushkevich P. A. (2010). N4ITK: improved N3 bias correction. IEEE Transac. Med. Imaging 29, 1310–1320. 10.1109/TMI.2010.2046908 20378467 PMC3071855

[B49] Van Horn J. D. Toga A. W. (2014). Human neuroimaging as a “Big Data” science. Brain Imaging Behav. 8, 323–331. 10.1007/s11682-013-9255-y 24113873 PMC3983169

[B50] Wells W. M. Grimson W. E. L. Kikinis R. Jolesz F. A. (1996). Adaptive segmentation of MRI data. IEEE Transac. Med. Imaging 15, 429–442. 10.1109/42.511747 18215925

[B51] Xu T. Yang Z. Jiang L. Xing X.-X. Zuo X.-N. (2015). A connectome computation system for discovery science of brain. Sci. Bull. 60, 86–95. 10.1007/s11434-014-0698-3

[B52] Zhao L. Ruotsalainen U. Hirvonen J. Hietala J. Tohka J. (2010). Automatic cerebral and cerebellar hemisphere segmentation in 3D MRI: adaptive disconnection algorithm. Med. Image Anal. 14, 360–372. 10.1016/j.media.2010.02.001 20303318

[B53] Zijdenbos A. P. Dawant B. M. Margolin R. A. Palmer A. C. (1994). Morphometric analysis of white matter lesions in MR images: method and validation. IEEE Trans. Med. Imaging 13, 716–724. 10.1109/42.363096 18218550

